# The Association between Pesticide Exposure and the Development of Fronto-Temporal Dementia-Cum-Dissociative Disorders: A Review

**DOI:** 10.3390/brainsci13081194

**Published:** 2023-08-12

**Authors:** Carlos Alfonso Flores-Gutierrez, Erandis Dheni Torres-Sanchez, Emmanuel Reyes-Uribe, Juan Heriberto Torres-Jasso, Mireya Zoila Reyna-Villela, Daniel Rojas-Bravo, Joel Salazar-Flores

**Affiliations:** 1 Department of Medical and Life Sciences, Centro Universitario de la Cienega (CUCI-UdeG), University of Guadalajara, Avenida Universidad #1115, Ocotlan 47810, Jalisco, Mexico; calfonso.flores@alumnos.udg.mx (C.A.F.-G.); erandis.torres@academicos.udg.mx (E.D.T.-S.); emmanuel.reyes@academicos.udg.mx (E.R.-U.); 2 Department of Biological Sciences, University Center of the Coast, University of Guadalajara (CUCos-ta-UdeG), Avenida Universidad de Guadalajara #203, Delegacion Ixtapa, Puerto Vallarta 48280, Jalisco, Mexico; heriberto.torres@academicos.udg.mx; 3 Department of Technological Sciences, Cienega University Center (CUCI-UdeG), University of Guadalajara, Avenida Universidad #1115, Ocotlan 47810, Jalisco, Mexico; mireya.reyna@academicos.udg.mx (M.Z.R.-V.); drojas@cuci.udg.mx (D.R.-B.)

**Keywords:** pesticides, acetylcholinesterase, Parkinson’s disease, free radicals, oxidative stress, dissociative disorders

## Abstract

Pesticides are chemicals used in agricultural fields for the prevention or destruction of pests. Inappropriate use of these substances, as well as handling them without using personal protective equipment, may result in serious health problems such as neurodegenerative diseases and mental disorders. Previous studies have demonstrated the adverse effects of pesticides on brain function. However, some researchers have associated pesticide poisoning with the development of disorders such as dissociative amnesia, multiple personality disorders, and depersonalization disorder. The objective of this work was to perform a bibliographic review of the relationship between pesticide poisoning and the development of dissociative disorders. Previous studies suggest that the duration of pesticide exposure is a major determinant in the development of dissociative diseases and disorders. The information obtained in this review suggests that there is no specific relationship between dissociative disorders and pesticide poisoning. However, these results point to associating the most representative symptoms of dissociative disorder (such as amnesia and memory loss) with pesticide exposure. Based on the bibliographic search, possible mechanisms of action were suggested in an attempt to explain a possible association between exposure to pesticides and the appearance of dissociative disorders.

## 1. Introduction

Pesticide poisoning represents a great risk to the health of the global population. A study carried out by the ONU in 2017 showed that 200,000 deaths from poisoning are recorded annually, especially in developing countries [1]. Farmers are highly vulnerable to pesticide poisoning due to direct contact with these chemicals. Among the most widely used pesticides in the world are organophosphates (OPs), carbamates, and pyrethroids [2]. Speaking specifically of OPs, worldwide it is estimated that around 3 million people are exposed to this type of pesticide each year, which represents approximately 300,000 deaths [3].The mechanism of action of OPs is through the inhibition of acetylcholinesterase (AChE) in the synapse, which leads to an accumulation of acetylcholine (ACh) from cholinergic receptors (muscarinic and nicotinic) in the central and autonomic nervous system and in the neuromuscular junctions, causing different clinical manifestations [4]. The degree of poisoning and the symptoms manifested depend on factors such as age, sex, the pathologies of the individual, type of pesticide, as well as dose and exposure time, which determine whether the poisoning will be classified as acute or chronic. An acute exposure is generated in a short time, generally between minutes and the first 24 h, with representative symptoms such as bronchorrhoea, vomiting, diarrhea, salivation, miosis, headache, and dizziness. In contrast, chronic poisoning occurs after a long period of exposure, and it causes damage to the neurological system, oxidative stress, and immune system symptoms [5]. In addition, OPs impair the functions of serotonergic and dopaminergic neurons and the expressions of genes related to neurobehavior. Thus, exposure to these pesticides affects the central nervous system, thereby causing behavioral disorders at different stages of human development. Alterations in neurotransmitter systems affect memory-related functions and damage neural connections, leading to neurodevelopmental disorders [6].

Although pesticides produce harmful effects on the brain by triggering severe illnesses such as Parkinson’s disease, Alzheimer’s disease, and Major Cognitive Disorder, not much information is available about the link between pesticides and the development of other mental illnesses such as frontotemporal dementia and dissociative disorders. Therefore, this study was carried out with the objective of doing a review of the literature to find a possible association between exposure to pesticides and the development of these mental disorders.

### 1.1. Mental Disorders and Pesticide Poisoning

#### 1.1.1. Frontotemporal Dementia (FTD)

Worldwide, it is estimated that fifty million people undergo dementia, a figure that has increased to ten million cases each year, ranking among the ten main causes of death, with a negative impact on the psychological, physical, and social level that affects the quality of life of patients and represents one of the main causes of dependency and disability [7]. The most relevant types of cortical dementias are dementia with Lewy bodies, Alzheimer’s disease, and FTD [8].

FTD is a neurodegenerative disease characterized by progressive changes in personality and behavior and/or early and progressive language impairment [9]. It is the second most common cause of dementia in people under 65 years of age [10]. Due to the heterogeneity of cognitive alterations, FTD comprises three clinical variants: behavioral/frontal, primary progressive aphasia, and semantic dementia [11]. These variants are shown in Table 1.

##### Front Variant

**Figure 1:**
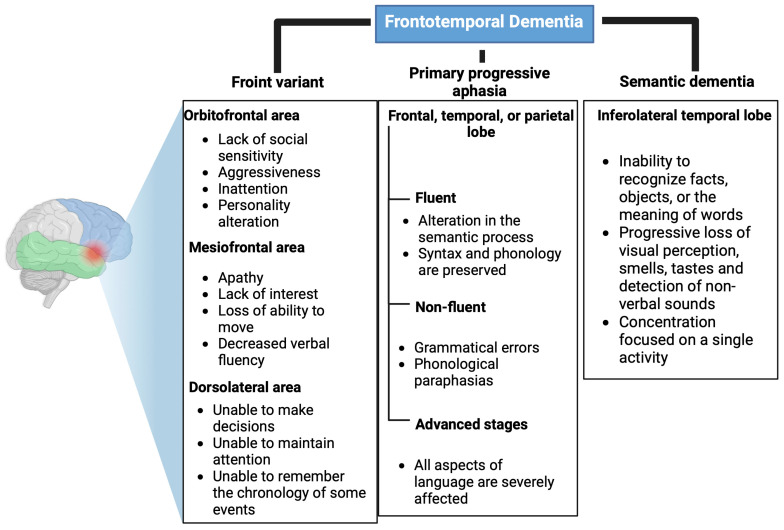
Clinical variants of frontotemporal dementia. Affected areas and main symptoms of the clinical variants of frontotemporal dementia: Front variant, primary progressive aphasia, and semantic dementia. Created with Biorender.com.

The frontal variant represents 90% of FTD cases, and it presents very varied alterations depending on the affected prefrontal area. If it affects the orbitofrontal area, it causes a lack of social sensitivity, aggressiveness, inattentiveness, and changes in personality. On the other hand, if the mesiofrontal area is affected, it results in apathy, a lack of interest, a loss of ability to move, and decreased verbal fluency. Finally, if the dorsolateral area is altered, patients are unable to make decisions, maintain attention, or remember the chronology of events [12,13]. These alterations are shown in Figure 1.

##### Primary Progressive Aphasia

In primary progressive aphasia, syntactic and phonological language alterations occur, which at the initial stages produce an inability to remember names of people or objects, followed by agrammatism, which may end in mutism [13]. This aphasic syndrome is divided into two variables: fluent (affects semantic processing, but syntax and phonology are preserved) and non-fluent, i.e., grammatical errors and phonological paraphasia [14,15]. In advanced stages of the disease, when all aspects of language are severely affected, memory deficits and behavioral changes may also manifest (Figure 1) [15].

##### Semantic Dementia

Semantic dementia is characterized by an inability to recognize facts, objects, or the meaning of words. In addition, the ability to perceive visual stimuli, smells, tastes, and non-verbal sounds is progressively lost. People with semantic dementia may present behavioral changes that are different from the frontal variant since they concentrate most of their time on a single activity in contrast to loss of interest, which is a characteristic of the frontal variant (Figure 1) [16].

##### Relationship FDT Variants with Pesticides

There are highly toxic pesticides for dopaminergic neurons [17]. The loss of dopamine in the connections between the prefrontal cortex and caudate nucleus leads to inhibition of response and planning. This results in the inability to sustain directed activities to achieve a set goal or objective and the inability to correctly perform motor actions associated with complex movement [18]. This may be associated with the frontal variant of FTD since if the mesiofrontal area is affected, there is loss of movement in the same way. An alteration in the dorsolateral zone leads to the inability to make decisions, which could explain the lack of planning and response due to a decrease in the level of dopamine in the connection between the prefrontal cortex and caudate nucleus [12,13,18]. It should be noted that the decrease in the amount of dopamine is also related to the use of chemical herbicides such as Paraquat, Diquat, and the fungicide MANEB, which are toxic to nigrostriatal dopaminergic neurons [17].

#### 1.1.2. Parkinson’s Disease (PD)

PD is the second most frequent neurodegenerative disease after Alzheimer’s disease [18]. The main signs of PD are akinesia, postural rigidity, and tremor at rest, which are the consequences of impairment in dopaminergic activity. Neuronal death, mainly in the substantia nigra, leads to a reduction in dopamine levels, which is responsible for the characteristic signs of the disease. In addition, the degeneration of the substantia nigra causes deficits in balance [19]. Epidemiological data suggest that there is an increased risk of developing PD after exposure to pesticides [20]. Pesticides such as paraquat, rotenone, and dieldrin induce apoptosis of dopaminergic neurons [21].

On the other hand, the presence of α-synuclein (a protein that forms Lewy bodies) in the enteric nervous system, submandibular gland, dorsal motor nucleus of the vagus nerve, and olfactory bulb may cause these peripheral areas to act as receptors for environmental factors that trigger the onset of PD, such as the pesticides rotenone and paraquat [18]. Heiko et al. (2003) described the progression of PD in 6 stages related to the presence of α-synuclein in different locations, and its clinical correlation. Stages 1–3 correspond to the preclinical phase of the disease. At these stages, α-synuclein aggregates are found at the level of the sympathetic and parasympathetic pre- and post-ganglionic fibers of the enteric nervous system connected to the olfactory bulb, dorsal nucleus of the vagus nerve, and at the level of the spinal cord. In stage 4, there is neurodegeneration in the zona pars compacta of the substantia nigra. Finally, in stages 5–6, the α-synuclein aggregates are distributed in sensory association areas corresponding to the premotor and motor prefrontal cortex [22].

Table 2 shows different pesticides related to the development of PD. Organochlorines (dieldrin and lindane) have been associated with PD. Various studies have shown the toxic effects of dieldrin and lindane on dopaminergic neurons, resulting in ROS-induced apoptosis of these neurons. In addition, postmortem studies have found high concentrations of organochlorine pesticides in the substantia nigra [21,23,24].

Animal studies have shown the involvement of organophosphates (OPs) in the development of PD. For example, chronic exposure to dichlorvos caused nigrostriatal dopaminergic degeneration and a 60–80% decrease in levels of tyrosine hydroxylase and dopamine in the striatum, while malathion increased the concentration of α-synuclein in the striatum. Moreover, OPs induce damage to the dopaminergic system through the production of ROS [24,25,26]. The pyrethroids cypermethrin and deltamethrin interfere with dopaminergic mechanisms involved in the production of dopamine, thereby decreasing the level of this neurotransmitter in the brain [27].

Several studies have demonstrated that paraquat is absorbed by the dopamine transporter, thereby resulting in toxicity and apoptosis due to oxidative stress [27,28,29]. Moreover, in an animal study, Drolet et al. (2009) reported that rotenone administration caused loss of striatal dopamine and accumulation of α-synuclein in the other dopaminergic neurons, in addition to the appearance of PD characteristics such as rigidity, postural instability, and bradykinesia [30].

**Table 2:** Pesticides associated with pathogenesis of PD.

Pesticide Classification	Type of Pesticide	Relationship with PD	Type of Study	References
Organochlorines	Dieldrin	Acted as dopaminergic toxin in mesencephalic cultures.	Review	[27]
		Reduced brain dopamine levels, increased ROS in nigral dopaminergic neurons, inhibited mitochondrial oxidative phosphorylation, altered mitochondrial membrane potential, and caused cytochrome C release.	Review	[21]
		There were increased concentrations of these insecticides in the substantia nigra, which may be directly related to reduced dopamine concentrations	Post mortem study	[23]
	Lindane	There were increased concentrations of these insecticides in the substantia nigra, which may be directly related to reduced dopamine concentrations	Post mortem study	[23]
	Others	They were neurotoxic, produced oxidative stress, and damaged the dopaminergic system.	Animal model	[24]
OPs	Dichlorvos	Produced nigrostriatal dopaminergic degeneration, reduced levels of striatal dopamine and tyrosine hydroxylase.	Animal model	[25]
	Malathion	Increased levels of α-synuclein protein and mRNA in striatal tissue	Animal model	[26]
	Others	Were neurotoxic, and produced oxidative stress and damage to the dopaminergic system	Animal model	[24]
Pyrethroids	Cypermethrin	Interfered with cholinergic and dopaminergic neurotransmission mechanisms	Review	[27]
	Deltamethrin	Reduced dopamine levels	Review	[27]
Others	Rotenone	Were selectively toxic to dopaminergic neurons	Human brain spheroid model from induced pluripotent stem cells	[31]
		Mitochondrial toxin	Case-control studies	[32,33]
		Reduced tyrosine hydroxylase-positive neurons in the substantia nigra, induced loss of striatal dopamine, and accumulation of α-synuclein and polyubiquitin-positive aggregates in the remaining dopaminergic neurons	Animal model	[30]
	Paraquat	Damaged dopaminergic neurons due to oxidative stress	Case-control studies	[27]
		Caused cellular toxicity due to oxidative stress to dopaminergic neurons.	Animal model	[28]
		Led to loss of dopaminergic neurons due to oxidative stress.	Review	[29]

#### 1.1.3. Dissociative Disorders

These disorders refer to mental disorders that involve disconnection and a lack of continuity of thoughts, memory, environments, actions, and identity. A person with a dissociative disorder is disconnected from reality in involuntary and unhealthy ways, leading to problems with daily functioning [34]. In the general population, the prevalence of dissociative disorders is 5% to 10%, while in psychiatric patients it is 10.2% to 41.4% [35]. Three main types of dissociative disorders are recognized: dissociative amnesia, dissociative identity disorder, and de-personalization/de-realization disorder [34].

##### Dissociative Amnesia

Dissociative amnesia (DA) is a disorder characterized by disruptions in the proper functioning of consciousness, memory, identity, and behavior. The affected person is unable to remember moments of their own life. In DA, there are alterations in brain sections such as the frontal cortex, hippocampus, temporal lobes (medial, right and left), hypothalamus, and thalamus. These structures are crucial in the processing of memory and emotional content, and they are activated in response to stress. However, certain neurotransmitters are also released, but in situations of minimal stress, the concentrations of dopamine, serotonin, acetylcholine, and norepinephrine are altered in the amygdala, hippocampus, and prefrontal cortex. Acute stress produces variations in levels of corticosterone, a hormone that regulates the release and activities of the aforementioned neurotransmitters and, under normal conditions, consolidates long-term memory. Consequently, changes in the action of corticosterone cause alterations in the activities of the different neurotransmitters. These changes are associated with an inability to remember events, the development of disruptive behaviors, and, ultimately, the development of DA [36,37].

##### Dissociative Identity Disorder (DID)

Formerly known as multiple personality disorder, it is a condition in which two or more different personalities coexist, with each one of them having its own criteria for perceiving and thinking about itself and the external environment. In this disease, there are serious changes in the way a person feels about himself, in addition to important alterations in behavior, memory, perception, cognition and functioning of sensorimotor [38].

Currently, it is more acceptable to refer to this condition as discontinuity in memory and identity. Dissociative identity disorder (DID) has the characteristic symptoms of other dissociative disorders (AD and de-personalization/de-realization). It is important to point out that for the diagnosis of DID, DA must be present. This explains the amnesia that the other personalities manifest. Patients with DID may present transient psychotic symptoms, e.g., hallucinations [39].

##### Depersonalization/De-Realization (DD)

This is a psychiatric condition characterized by permanent feelings of unreality about the outside world as well as self-detachment. Depersonalization is an experience in which the person affected does not control his thinking. Consequently, he feels alienated, distant, and unable to identify himself. In contrast, de-realization is the feeling of strangeness towards the outside world. Both experiences are related. However, the patient may suffer episodes of de-realization without having experienced de-personalization, and vice versa. Due to their inability to describe experiences clearly, these patients often resort to metaphors, and since they never lose contact with reality and are able to identify sensations during DD episodes, this disorder is considered a type of neurosis [40,41]. Depersonalization may be due to alterations in the levels of different neurotransmitters, for example, glutaminergic N-methyl-D-aspartate (NMDA) receptors distributed throughout the amygdala, hippocampus, and cerebral cortex. These neurotransmitters are involved in associative functions in long-term memory. In cholinergic overstimulation, the activation of these receptors induces the release of nitric oxide, which causes neurotoxicity and neuronal degeneration by interacting with the superoxide radical to form nitric superoxide [42,43].

**Figure 2:**
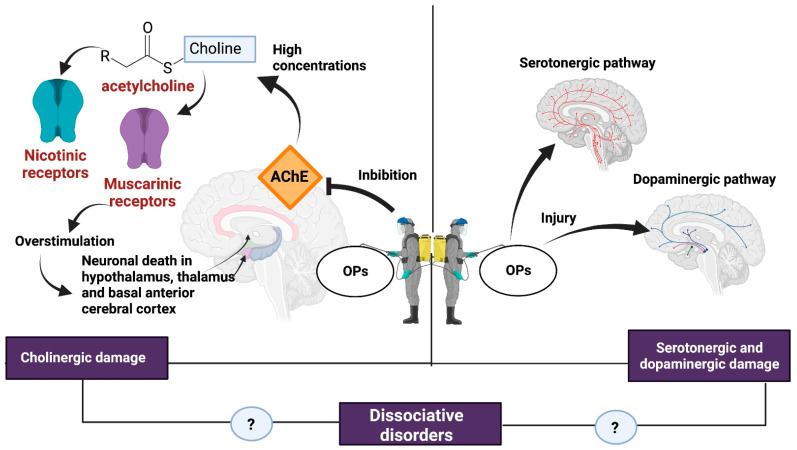
Damage caused by OPs. some of the consequences of exposure to OPs such as cholinergic syndrome and serotonergic and dopaminergic damage could be related to the development of dissociative disorders. Created with Biorender.com.

The neurotoxicity of some types of pesticides affects the synthesis, release, absorption, and degradation of different neurotransmitters. The OPs affect the central nervous system by inhibiting AChE, which catalyzes the hydrolysis of ACh into choline and acetate. Because of AChE inhibition, an excessive accumulation of ACh occurs in the synaptic cleft, causing overstimulation of nicotinic and muscarinic receptors located both in the central nervous system and at the neuromuscular junction, hence the importance of the dose, exposure time, and severity of the poisoning [6,44] (Figure 2).

The overstimulation of muscarinic receptors results in a loss of integrity of the cholinergic pathways and generates neuronal death in brain regions such as the hypothalamus, thalamus, and basal anterior cerebral cortex [6]. Stimulation of muscarinic receptors causes miosis, urination, bradycardia, bronchorrhea, bronchospasm, emesis, and salivation [45].

Overactivation of the nicotinic receptor favors the influx of Ca^2+^ into the cell and promotes increased levels of reactive oxygen species (ROS), oxidative stress, and apoptosis [46]. In addition, the production of ROS in the mitochondria causes the release of cytochrome C, which in turn activates caspases 3 and 9, increasing the expression of apoptotic genes [47]. At the central nervous system level, nicotinic effects cause headaches, confusion, anxiety, ataxia, and seizures [45].

OPs also inhibit the esterase target of neuropathy, present in peripheral nerves, lymphocytes, and the brain, causing tardive polyneuropathy [48]. Conditions seen in subjects with tardive polyneuropathy include depression, psychosis, and impaired processing and problem-solving abilities [49].

A recent study sought to associate OP exposure with neurological disorders such as dementia, attention deficit hyperactivity disorder, Parkinson’s, cognitive development, chronic neuropsychiatric disorders, and autism. The authors presented evidence of a strong link between repeated exposure to OPs and neurodevelopmental, cognitive, and attention deficit disorders in children [46].

## 2. Discussion

Although the existing information on a possible relationship between the development of dissociative disorders and pesticide exposure is null, an association can be made between the characteristic symptoms of said disorders, particularly dementia and memory loss since there is evidence that the pesticides participate in the development of these symptoms, affecting different neurotransmitters or organs, and may represent a risk factor acting alone or in conjunction with other components such as stress, abuse, or a congenital disease related to the development of dissociative disorders.

The main alterations in different organs, organelles, and neurotransmitters due to pesticide exposure are discussed in Table 3. The mechanisms involved in these alterations are similar to the mechanisms that trigger characteristic symptoms of dissociative disorders, mainly amnesia and loss of memory.

One of the mechanisms involved is the one that induces rotenone by inhibiting the electron transport chain in the mitochondria, causing hyperphosphorylation of the Tau protein, the formation of β-amyloid peptides, and increased lipid peroxidation (causing oxidative stress). DDT also impairs the electron transport chain. [50,51,52,53].

Likewise, Souders et al. (2021) reported malformation of the nervous system, neurofibrillary tangles, neuroinflammation, and nerve fiber degeneration in neuronal cells of rats exposed to fipronil [54]. Moreover, Cam et al., (2018) have demonstrated that fipronil stimulated the production of amyloid peptides which constitute a characteristic abnormality in Alzheimer’s disease [55].

Pesticides such as dieldrin, rotenone, and paraquat induce apoptosis of dopaminergic neurons, thereby altering their proper functioning in the brain and resulting in neurodegenerative conditions such as Parkinson’s disease and/or Alzheimer’s disease [21].

OPs are AChE inhibitors that impair the cholinergic system and interrupt axonal transport and mitochondrial function, thereby triggering neurological disorders, e.g., memory loss [44,56]. OPs are distributed to all regions of the brain, including the hippocampus, the major region involved in learning and memory regulation. Organophosphate-oxons disrupt the development of the nervous system, glial cell proliferation, differentiation, and neural function [57]. Other evidence indicates that there is a gradual loss of neuronal cells in the amygdala in acute diisopropylfluorophosphate poisoning, with behavioral changes as the major consequence [58]. Constant memory deterioration is one of the most observed behavioral changes in people intoxicated with OPs [59].

Glyphosate, a broad-spectrum herbicide, causes oxidative stress-induced neuronal alteration [60].

A study by Nasuti et al. (2014) showed that early exposure to permethrin led to severe hippocampal lesions such as impairment of long-term memory and disruption of synaptic morphology [61]. Continued exposure to deltamethrin, a synthetic pyrethroid, led to apoptotic cell death in the hippocampus, endoplasmic reticulum stress, and decreased proliferation of the hippocampal precursor, all of which are associated with learning deficits [62].

It has been reported that chronic administration of chlorpyrifos significantly impaired learning, memory, and motor coordination [63]. Exposure to pesticides such as parathion, hexachlorocyclohexane, and aldrin resulted in an increased risk of dementia and the subsequent onset of Alzheimer’s disease [64]. Chronic exposure to OPs and organochlorines produces neurobehavioral effects [65].

**Table 3:** Organelles/organs/neurotransmitters altered by pesticides, and their association with dissociative disorders.

Organelle/Organ/Neurotransmitter	Pesticide	Mechanism	Effects Related to DA, DID and DD	Reference
Mitochondria	Rotenone	Inhibition of the electron transport chain	Production of mitochondrial and neuronal ROS, resulting in neuroinflammation	[52]
	DDT	Alteration oxidative phosphorylation		
GABA	Fenilpirazol fipronil	Blockage of ionotropic γ-aminobutyric acid (GABA) receptors in the central nervous system	Central nervous system overexcitation, seizure, and death	[54]
Dopamine		Stimulation of the production of amyloid peptides Aβ42/Aβ43	Alzheimer’s disease	[55]
Dopamine	Dieldrin	Crossing of the blood-brain barrier, selective toxicity to dopaminergic neurons	Apoptosis of dopaminergic neurons	[21]
	Rotenone	Mitochondrial toxicity due to production of ROS		
	Paraquat	Production of ROS when taken up by the dopamine transporter		
Acetylcholine	OPs	AChE inhibition	Alteration of the cholinergic system	[44]
Mitochondria/axons		Disruption of mitochondrial function and axonal transport	Neurological disorders	[56]
Cell membranes	Rotenone	Exacerbation of lipid peroxidation	Oxidative stress	[53]
Mitochondria	Glyphosate	Loss of the active site for oxidative phosphorylation	Oxidative stress and inhibition of oxidative phosphorylation	[60]
Hippocampal	Permethrin	Hippocampal dysfunction and amygdala impairment	Panic disorder, alteration in the morphology of the hippocampus and attention deficit	[61]
Hippocampal ER	Deltamethrin	Apoptosis in SK-N-AS neuroblastoma cells, increased levels of C/EBP homologous protein, glucose-regulated protein 78 caspase-12, activated caspase-3, and decreased BrdU-positive cells	Stress, learning deficits, and impaired hippocampal neurogenesis	[62]
Hippocampus	Paraquat	Alteration of levels of the Wnt pathway genes in neural progenitor cells	Oxidative stress and inhibition of cell viability and proliferation	[66]
Dopamine	Rotenone	Alteration of levels of the Wnt pathway genes in neural progenitor cells	Changes in locomotor behavior and alteration in gene expression in dopaminergic neurons	[67]
Brain	Chlorpyrifo	AChE inhibition	Impaired memory and motor function	[63]
	OPs	AChE inhibition	Brain damage from cholinergic neuronal dysfunction and excitotoxicity	[65]

Table 4 presents specific evidence of the association between pesticides and dissociative disorders.

In DA, there is an alteration in brain structures, with high levels of pesticides such as glufosinate ammonium accumulating in the brain, thereby affecting the central nervous system and producing alterations in adults [68]. Studies have shown that parathion causes alterations in cholinergic, serotonergic, and dopaminergic synaptic functions in brain regions. Changes in neurotransmitter systems alter memory-related functions and affect neural connections, thereby contributing to the formation of neurodevelopmental disorders [6,69,70].

Dissociative Identity Disorder (DID) is associated with behavioral changes, memory loss, and amnesia. Evidence for the link between these symptoms and pesticides, particularly OPs, was found in medical studies carried out on patients with prolonged pesticide exposure who manifested chronic changes in behavior such as personality alteration, decreased memory, anxiety, depression, nervousness, and incoordination [71,72]. García (2000) carried out a descriptive follow-up study of patients with a history of acute poisoning by OPs. The main symptoms found corresponded to changes in behavior, such as irritability, difficulty with concentration, depression, memory problems, and tiredness [73].

Finally, dieldrin is a GABA A receptor antagonist that has been shown to decrease the number of functional NMDA receptors in primary cultures of mouse cortical neurons. Since these receptors are involved in associative functions in long-term memory, a decrease in their activity produces symptoms of DD [43,74].

**Table 4:** Pesticides that modify neurological functions related to dissociative disorders.

Disorder	Neurological Alteration	Pesticide	Reference
DA	Alteration in frontal cortex, temporal lobes and diencephalon. Changes in levels of dopamine, serotonin, ACh and norepinephrine	Glufosinate ammonium Parathion	[6,68]
DID	Peripheral neuropathy and behavioral disturbance	OPs	[73]
DD	Intracellular accumulation of calcium through stimulation of anti-N-methyl-D-aspartate (NMDA) receptors and ROS production	Dieldrin OPs	[43,74]

## 3. Conclusions

Exposure to different types of pesticides could be related to neurotransmitter, organelle, and effector organ dysfunction that triggers dissociative disorders. These types of disorders are becoming more frequent in human populations, as is the massive use of different pesticides; however, this review exposes the necessity to increase the number of prospective studies that evaluate the neurotoxic potential of these substances and their relationship with dissociative disorders in humans.

The information shown in this review suggests that exposure to organochlorines, OPs, pyrethroids, etc., produces different brain disorders such as Alzheimer’s disease and PD. At the same time, it opens the possibility of developing new lines of research focused on exposure to pesticides and dissociative disorders. This possibility could provide alternatives that contribute to the prevention and treatment of these disorders.

However, more research is necessary to link pesticide exposure to the pathophysiology of dissociative disorders in humans. In addition, a larger number of studies are required in subjects exposed to a single pesticide to associate its effects with the dysfunction of different neurotransmitters, organelles, and effector organs, since the analysis of the data in this review comes from studies performed mainly in animals.

Finally, the analysis of the data shows the relationship between pesticides and symptoms of dissociative disorders, not those resulting from pesticide exposure, and does not describe the molecular mechanisms involved.

## Figures and Tables

**Table 1:** Clinical variants of FTD.

Variant	Symptomatology	Anatomical Lesion
Behavioral/frontal	Changes in personality and behavior (loss of emotionality, increased consumption of sweet foods, lack of interest in activities previously carried out, and neglect of personal care)	Bilateral orbitofrontal cortex
Primary progressive aphasia	Non-fluent aphasia, altered. expression, but preserved comprehension.	Perisylvian area
Semantic dementia	Fluent anomic aphasia with impaired comprehension and loss of meaning.	Bilateral or left inferolateral temporal cortex

Modified from [12].

## Data Availability

Data are available on request from the authors.
